# An unusual case of proximal humeral simple bone cyst in an adult from secondary cystic change

**DOI:** 10.1186/s12957-017-1166-8

**Published:** 2017-05-15

**Authors:** Mamer S. Rosario, Norio Yamamoto, Katsuhiro Hayashi, Akihiko Takeuchi, Hiroaki Kimura, Shinji Miwa, Takashi Higuchi, Hiroyuki Inatani, Kensaku Abe, Yuta Taniguchi, Hisaki Aiba, Hiroyuki Tsuchiya

**Affiliations:** 1 Department of Orthopaedic Surgery, Kanazawa University Graduate School of Medical Sciences, 13-1 Takara-machi, Kanazawa, 920-8640 Ishikawa Japan; 2 Department of Orthopaedics, East Avenue Medical Center, East Avenue, Diliman, 1101 Quezon City, Metro Manila Philippines

**Keywords:** Case report, Simple bone cyst, Unicameral bone cyst, Solitary bone cyst, Secondary cystic change, Trauma

## Abstract

**Background:**

Simple bone cysts (SBC) have been documented to occur in adults with closed physeal plates, most commonly affecting the calcaneus in this patient subset. Although most authors theorize an association to trauma, etiology of simple bone cysts remains an enigma up to now.

**Case presentation:**

A 26-year-old kickboxing coach sought consult for a painful right shoulder which on radiographs and magnetic resonance (MR) imaging showed a proximal humeral lesion with signs of ossification. The patient was lost to follow-up but again sought consult after 3 years for the recurring complaint. On repeat radiographs, computed tomography (CT) scan, and MR images, tumor enlargement with cystic findings typical of simple bone cyst were documented. Diagnostic aspiration of the lesion was firstly done, revealing straw-colored fluid. The patient then underwent intralesional curettage with alpha-tricalcium phosphate cement reconstruction of the lytic defect. No perioperative complications were incurred, and on latest follow-up at 3 years postoperatively, Musculoskeletal Tumor Society (MSTS) and visual analog scale (VAS) pain scores were 30/30 and 0/10, respectively.

**Conclusions:**

The authors believe their report provides support to a possible association to trauma of simple bone cysts occurring in the adult population with closed physes and suggest this subset of patients may require a different treatment approach from that for juvenile simple bone cysts.

## Background

The etiology of simple bone cyst (SBC) remains inconclusive [1], although the lesion seems to be dysplastic or reactive rather than a true tumor [2]. Theories being proposed include local disturbances in bone growth [3], pressure effects due to blocked fluid drainage [4], local venous obstruction [5], nitric oxide [6], increased lysosomal enzyme activity [7], prostaglandins [8], oxygen free radicals [9], disorders of synovial origin [10], and genetic causes [11]. Cases of SBC following trauma have been observed, theorized to be forming consequently from intraosseous hemorrhage when mechanisms of bone organization and repair fail [12, 13]. The latter are an entity separate from the unconventionally described pseudocyst forming in periprosthetic tissues or following a fracture or minor trauma [14], which histologically resembles a synovial cyst with synovium-like lining with or without a single-layered epithelium [15]. The enigma answers why different methods of treatment have been reported to resolve the disease, all of which remain controversial [1, 16].

Huch et al. mentioned the term “solitary bone cyst” to connote a SBC occurring in an adult, as opposed to “juvenile bone cyst” that occurs in the young population [17]. This must be remarkable, for SBC is described to be a lesion occurring in the first two decades of life [18–20] and graded as active or latent depending on the distance of the lesion to the physeal plate [21]. The recognition that SBC can occur in the adult [15, 17, 22], which is atypical, may possibly indicate a different pathogenesis. More so, the observation that most adult patients have calcaneally located SBC [22] lends the suggestion that in this subset of patients, association to trauma could be the likely etiology. The calcaneus endures concentration of forces through the heel, possibly developing intraosseous hematoma that subsequently liquefies to become SBC [23].

We hereby report a case of a solitary medullary lesion in the proximal humerus of a 26-year-old kickboxing coach, with signs of ossification on radiographs that, after 3 years, has atypically possessed radiological features of a SBC. SBC mainly involves the long bones especially the proximal humerus, accounting for about 51% of cases, and the most recurrences following ablation [24]. Although proximal humeral SBC seems to be common, the peculiarity of our case lies in its unusual clinical presentation, which, to the best of our knowledge, has no similar reports in the literature. We believe this report may add basis to suggest that in adults, and separate from younger patients, SBC presents a “trauma-hemorrhage” etiopathogenesis.

## Case presentation

**Fig. 1:**
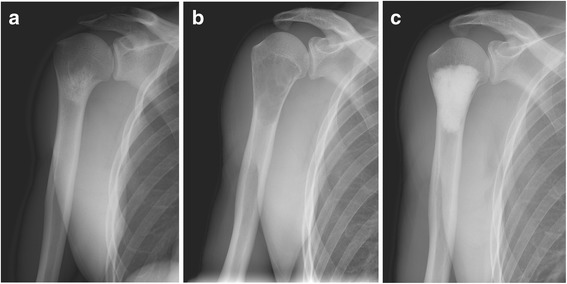
**a**–**c** AP views of the right shoulder. **a** initial presentation, showing a sclerotic medullary lesion in the proximal humerus with foci of calcifications. **b** At 3 years later, showing enlargement of the tumor with endosteal thinning and lucent lytic changes typical of simple bone cyst. **c** After intralesional curettage and reconstruction of the lytic defect using *Biopex*

**Fig. 2:**
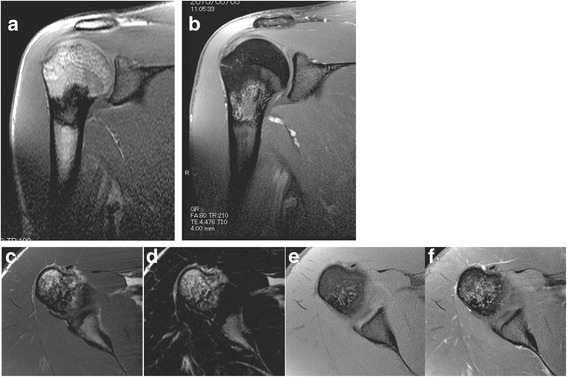
**a**–**f** MRI scans of the right shoulder at initial presentation. T1-weighted coronal (**a**), T1-weighted transverse (**c**), and T2-weighted fat-suppressed (**e**) images of the proximal humeral lesion showing primarily low to intermediate signal intensities with some small foci of high signal intensity. T2-weighted coronal (**b**), T2-weighted transverse (**d**), and with gadolinium contrast (**f**) images of the proximal humeral lesion showing primarily high signal intensities with some streaks of low signal intensity

A 26-year-old male with unremarkable past medical, family, and psychosocial history sought consult for a painful right shoulder at the previous hospital. Plain radiographs revealed a medullary sclerotic lesion in the right proximal humerus with foci of calcifications (Fig. 1a). On magnetic resonance (MR) imaging, the lesion showed primarily low to intermediate signal intensities with some small foci of high signal intensity on T1-weighted (Fig. 2a, c) and T2 fat-suppressed (Fig. 2e) sequences and primarily high signal intensities with some streaks of low signal intensity on T2-weighted (Fig. 2b, d) and contrast-enhanced (Fig. 2f) sequences. The patient was advised watchful observation of the lesion; however, the patient was eventually lost to follow-up.

**Fig. 3:**
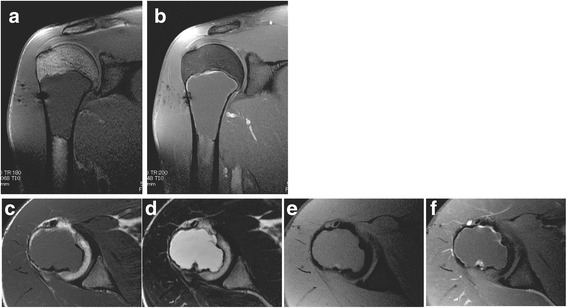
**a**–**f** MRI scans of the right shoulder at 3 years after initial presentation. T1-weighted coronal (**a**), T1-weighted transverse (**c**), and with gadolinium contrast (**f**) images of the proximal humeral lesion showing a homogenous low signal intensity. T2-weighted coronal (**b**), transverse (**d**), and fat-suppressed (**e**) images of the proximal humeral lesion showing a homogenous high signal intensity

After 3 years, the patient sought consults at the authors’ hospital, again for pain on his right shoulder. He admitted to sustaining hits to his right arm and shoulder during sparring, although other alleged information when he was lost to follow-up were unremarkable. Limited range of motion was observed, although no signs of deformity or inflammation were noted on the right shoulder. Plain radiographs revealed the lesion had enlarged, transforming to a well-defined, geographic, radiolucent lesion with endosteal thinning but no periosteal reaction (Fig. 1b). No fracture was detected on computed tomography (CT) scan. MR images showed a homogenous low signal intensity lesion on T1-weighted (Fig. 3a, c) and contrast-enhanced sequences (Fig. 3f), but a high signal intensity one on T2-weighted sequences (Fig. 3b, d, e). This lesion was diagnosed as simple bone cyst from the imaging studies.

**Fig. 4:**
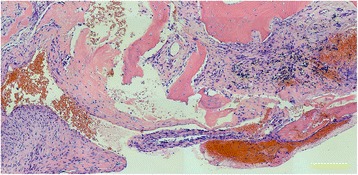
Final histopathological slide (hematoxylin and eosin stain; scale bar = 200 μm) showing clusters of the “amorphous” pink fibrinoid substance typical of a simple bone cyst, with scattered fibroblasts, fragments of bone tissue, and hemosiderin deposits

At the operating room, with the patient’s informed consent, the authors performed diagnostic aspiration of the lesion before the surgical incision, revealing straw-colored fluid and thereby confirming a working diagnosis of simple bone cyst. The authors proceeded with thorough intralesional curettage of the lesion, sampling of curetted tissue for final histopathological analysis, and reconstruction of the lytic defect (Fig. 1c) using alpha-tricalcium phosphate filling paste (*Biopex*, HOYA Technosurgical Co., Ltd., Tokyo, Japan). Final histopathological examination revealed “amorphous” pink fibrinoid substance typical of a simple bone cyst (Fig. 4).

Postoperatively, the patient had full active range of shoulder motion on the affected side by the first week, with no wound complications or infection. Follow-up radiographs showed no progression of the lesion. Latest follow-up at 3 years postoperatively revealed Musculoskeletal Tumor Society (MSTS) and visual analog scale (VAS) pain scores of 30/30 and 0/10, respectively.

## Discussion

SBC is a benign lesion that mostly affects children and adolescents and represents about 3% of primary tumors in the first two decades of life [18–20]. It is also known as solitary, unicameral, or traumatic bone cyst and, radiographically, is seen as a mildly expansile, lytic thin-walled bone lesion without periosteal reaction [20]. The typical location is in the metaphysis adjacent to the growth plate of the tubular bones, mostly the proximal humerus and femur [20]. Based on the distance between the lesion and the physis, SBC is classified as in active phase when the distance is less than 5 mm and latent when larger than 5 mm [21]. Other locations of this lesion may include the pelvis, ribs, vertebrae, and the tarsal bones, especially the calcaneus. Several theories have been postulated to explain the etiology of SBC including trauma and inflammation, but none has been conclusive [1–13].

It is important to note that in the above case, the lesion and its accompanying symptoms were documented in the proximal humerus of a 26-year-old kickboxing coach. The lesion occurred at an age past physeal plate closure and in a location most likely hit many times with “kicks” from sparring sessions during training. Presenting during the third decade of life, the simple bone cyst documented in this report is definitely atypical. And what is interesting is, 3 years prior to its formation, a different radiographic picture of the same lesion was apparent.

Major differential diagnoses for a similarly located lesion in a young adult include aneurysmal bone cyst, monostotic fibrous dysplasia, enchondroma, and eosinophilic granuloma [25, 26]. All of these lesions may be radiolucent on plain x-rays. However, clinical features typically associated with each of these lesions help differentiate these other diagnoses from SBC [27].

SBC arising from healed or healing fractures have been reported in the literature [14, 28] and described to affect a wider age group that included adults [15, 28]. Also, calcaneal SBC has been observed similarly [22]. Repeated micro-stresses are inherent to the calcaneus’s role of bearing weight, and so the “trauma-hemorrhage theory” suits well even with calcaneal SBC. SBC of the jaw, a rare variant of SBC [29], has been postulated to develop from multiple micro-traumas undergone by the teeth and alveolar processes [30]. Despite the myriad of theories presented in literature, SBC etiology is not clear enough. Harnet et al. [13], in their review of prevailing etiopathogenetic hypotheses for SBC, have found the “trauma-hemorrhage” theory the most widely accepted by authors. But some have questioned this mechanism, observing there is no history of trauma in more than 50% of cases reported, and postulated a multifactorial etiology instead [13, 31].

Neutrophils are the primary immune cells involved in acute inflammatory responses to trauma [32], acting as major sources of prostaglandin E2 (PGE2) [33]. Interleukin-1 (IL-1) has been demonstrated in vitro to induce osteoclast formation by a mechanism involving PGE2 [34, 35]. With biochemical analyses of SBC fluid having demonstrated increased levels of PGE2 [8, 36], IL-1 [36], proteolytic enzymes [7, 36], and acid phosphatase [37], we can now possibly link trauma-induced inflammation with SBC developing from an increased osteoclast activity. Having demonstrated the promotion of osteoblastic growth and differentiation with SBC fluid, Aarvold et al. proposed a receptor activator of nuclear factor kappa-B ligand (RANKL) signaling mechanism for the osteoclastogenesis [38], supporting an earlier study observing direct interaction between osteoclast progenitors and osteoblastic cells in the osteoclast recruitment induced by IL-1 [34].

The authors, from their experience in this report and review of the literature, hypothesize that a different etiopathogenesis could be serving as the basis for SBC formation in the adult bone, particularly a “trauma-associated” one. Peculiar to this report is the radiologic documentation of the precedent lesion 3 years prior to the apparent SBC formation. The initial radiographic features could actually be the picture of a posttraumatic medullary hematoma that subsequently liquefied later on to appear as a cyst 3 years later. This hypothesis by the authors also implies that a separate treatment approach may be needed for the adult-onset SBC. General treatment options reported in the literature include curettage in combination with autologous or allogenic grafting, the use of bone substitutes, autologous bone marrow injection, and numerous methods of cyst decompression including the use of cannulated screws or a cannulated hydroxyapatite pin [39–43]. The lack of a clear pathoetiology has impeded the development of a standard of care for solitary bone cysts amidst the numerous treatment strategies reported [1, 16, 40, 44]. With supporting evidence for a “trauma-hemorrhage” theory underlying SBC formation in the adult bone, surgical strategies, whenever necessary [1], may possibly be “downstaged” to minimally invasive ones, like prophylactic closed intramedullary nailing or bridge plating techniques for the diaphyseal lesion in a long bone or percutaneous reconstruction with grafts or bone substitutes as reported by some authors [42, 44, 45]. This is because, as illustrated by the theory, the traumatic cyst forms from a reactive process creating a defect [13] and not the “formation” process by an active epithelium lining [6]. For the adult-onset SBC, definitely when the “trauma-hemorrhage” theory is proven correct, thorough intralesional curettage may no longer be necessary.

## Conclusions

The authors hereby conclude that their case provides support to a possible association to trauma of solitary bone cysts occurring in the adult population and suggest this subset of patients may require a different treatment approach from that for juvenile simple bone cysts.

## Abbreviations

CT: Computerized tomography
MR: Magnetic resonance
MSTS: Musculoskeletal Tumor Society
SBC: Simple bone cyst
VAS: Visual analog scale
